# Nonlinear Electrical Properties and Field Dependency of BST and Nano-ZnO-Doped Silicone Rubber Composites

**DOI:** 10.3390/molecules23123153

**Published:** 2018-11-30

**Authors:** Juyi Guo, Xilin Wang, Zhidong Jia, Jun Wang, Chuan Chen

**Affiliations:** 1 Department of Electrical Engineering Graduate school at Shenzhen, Tsinghua University, Shenzhen 518055, China; gjy_one_thu@163.com (J.G.); jiazd@sz.tsinghua.edu.cn (Z.J.); 2 China National Electric Apparatus Research Institute Co., Ltd., Guangzhou 510080, China; wangjun@cei1958.com (J.W.); chenchuan@cei1958.com (C.C.)

**Keywords:** nonlinear filler, silicone rubber composites, dielectric properties, resistivity, temperature

## Abstract

Recently, composite materials with nonlinear dielectric or resistive properties performed well in electric field homogenization and space charge suppression in a high voltage transmission and distribution system. For the purpose of obtaining insulation materials with desirable dielectric and electrical resistance properties, we investigated several fillers with nonlinear electrical properties doped in silicon rubber composites, and their dependency on the temperature and field. The samples of silicone rubber composites with different components were prepared using barium strontium titanate (BST) and zinc oxide (ZnO) as the filler, and high temperature vulcanized silicone rubber (SiR) as the matrix. The investigations revealed that the BST-doped samples showed different dielectric properties compared to ZnO-doped composites, with an increase in the electric field, which was nonlinear. The resistivity of both doped samples was similar. Results demonstrated that it was possible to achieve higher values of permittivity, and lower values of tanδ and resistivity, with respect to unfilled silicone rubber composites over a wide electrical field and temperature range. Discussion of the results attributes these important functional behaviours to the spontaneous polarization of nonlinear nanoparticles and the interaction between the SiR chains and the nonlinear nanoparticles at the interfacial area.

## 1. Introduction

Silicone rubber material is widely used in high-voltage power transmission systems as an insulation material because of its desirable electrical insulation properties. But an extreme non-uniform electric field will result in the acceleration of partial electrical ageing of such polymer dielectric material, which is a vital threat to the long-term safe and stable operation of the power system. More importantly, with the rapid development of high voltage direct current (HVDC) flexible transmission technology, HVDC flexible transmission has gradually become the main transmission mode of large-scale new energy access, urban AC-DC hybrid power supply and marine power supply. Compared to a high voltage AC cable, a high voltage DC cable is more likely to lead to space charge one-way accumulation. This is due to the role of DC voltage, which contributes to the more severe problem of uneven distribution of the electric field and is a severe challenge for the insulation and electric field grading capacity of the insulation materials used in the high voltage DC cable terminations and joints, where the electric stress is critical. Recently, the prospect of using composites with nonlinear dielectric or electrical resistance properties, as smart materials for stress control and field grading in all fields of electrical insulation, is gaining attention due to the reported advantages of such functional materials with respect to their property of self-adapting matching between the material performance parameter and the space electric field strength.

A nanocomposite system with zinc oxide (ZnO) as the filler material has been widely investigated for its dielectric properties. Results have illustrated that ZnO filled composites exhibit prominent nonlinear conductivity and dielectric properties [1]. BaTiO_3_ was also the main filler in several composites materials, such as polymethyl methacrylate (PMMA), polyethylene (PE), epoxy and polyvinyl chloride (PVC), in order to enhance the dielectric permittivity of materials [2,3,4]. A lot of research has been done on Ba_x_Sr_1_-xTiO_3_ (BST) (0 ≤ x ≤ 1) nanoparticles and ceramics, due to their high dielectric constant and tunability (dependence of the dielectric constant on an applied electric field), but there are few reports on BST-doped composite materials. Considering the unique dielectric properties of BST particles and their dependency on the electrical field and temperature resulting from its ionic polarization, it is possible that BST-doped composites have a potential application in high voltage insulation materials, especially for non-linear field grading materials (FGM) [5]. As a result, we felt the need to examine the dielectric and electrical conductivity properties of BST-doped silicone rubber (SiR) nanocomposite systems as a function of the electrical field and temperature. The electrical properties of ZnO-doped SiR composites were also investigated as a comparison. Furthermore, an attempt was made to tailor the electrical properties of SiR composites by mixing ZnO and BST particles as the filler. The result verified the prospect of obtaining SiR composites with specific electrical properties by controlling the components of fillers.

To demonstrate the mechanisms leading to the obtained nonlinear dielectric properties, the spontaneous polarization of nonlinear nanoparticles, the interaction between nanoparticles and SiR chains and the formation of the interface region in the composite has been analyzed with the help of an interface model. As a continuation of the discussion of the interface model from the earlier publication, this paper correlated the interface model with the multi-core model based on the characteristics of the interfaces at both the interfacial layers [6]. An attempt was also made to correlate the interface characteristics with the dielectric and conductive properties obtained for SiR composite material.

## 2. Results

### 2.1. Conductivity Behaviors

**Figure 1:**
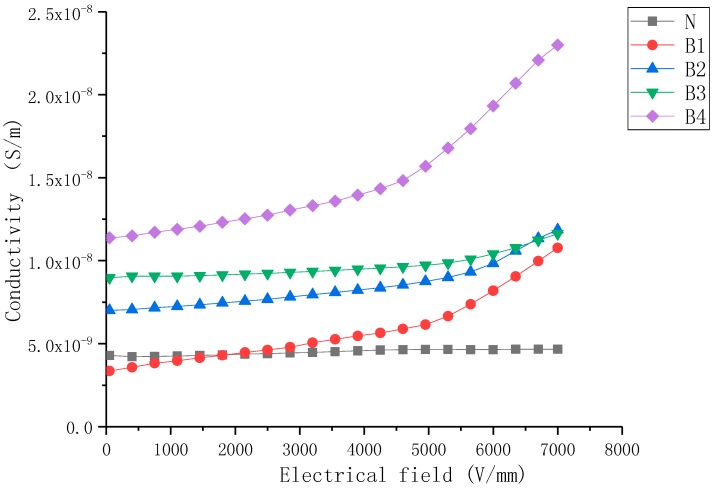
Conductivity characteristics of barium strontium titanate (BST)-doped silicone rubber (SiR) composite samples of different filler concentration at 25 °C.

**Figure 2:**
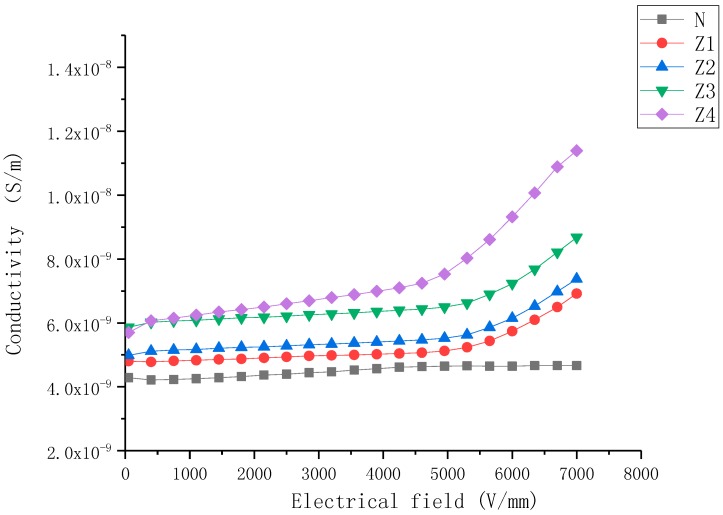
Conductivity characteristics of zinc oxide (ZnO)-doped silicone rubber (SiR) composite samples of different filler concentration at 25 °C.

**Figure 3:**
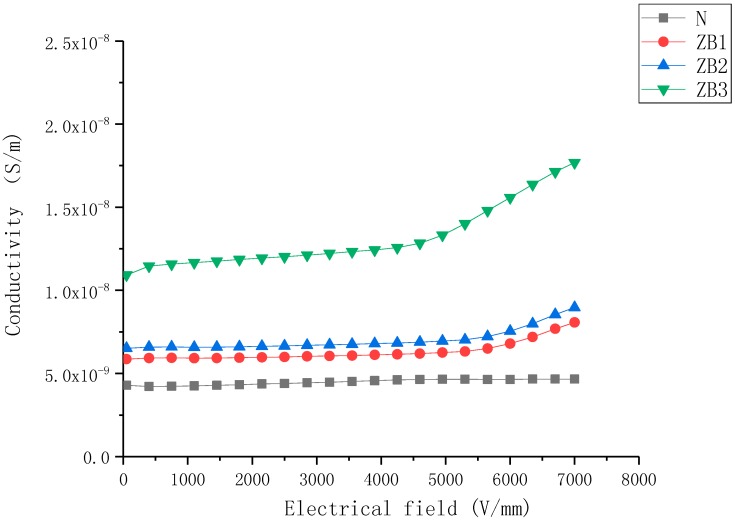
Conductivity characteristics of barium strontium titanate (BST) and zinc oxide (ZnO) doped silicone rubber (SiR) composite samples of different filler concentration at 25 °C.

The J–E characteristics of nonlinear nanoparticle-doped composite samples with filler content from 5 wt% to 30 wt% are shown in Figure 1, Figure 2 and Figure 3. 

Figure 1 presents the conductivity characteristics of BST-doped SiR composite samples as a function of the electric field. It can be noticed that the conductivity of samples increased with the increase in mass fraction of the BST fillers. The conductivity of the undoped SiR sample was almost the same with variation in the electric field. Sample B4 exhibited non-linear conductive behavior at high electric field values (Eb = 5kV/mm), whereas samples B1-3 exhibited it at relatively lower electric field values (Eb= 6 kV/mm). When E ≤ Eb, the conductivity of BST-doped samples slightly increased with an increasing electric field, and the variation was similar to that of undoped SiR samples. When E > Eb, the conductivity of BST-doped samples increased rapidly with the increase in E.

As shown in Figure 2, the conductivity characteristics of the ZnO-doped samples were very similar to the BST-doped samples in several ways: (a) the conductivity of samples increased with the increase in mass fraction of fillers, (b) when E ≤ Eb, the conductivity of samples changed little with field strength. When E > Eb, the conductivity of ZnO-doped samples increased rapidly with the increase in E, (c) the nonlinearity conductive coefficient (αj) increased as the mass fraction of fillers increased. However, some differences could still be observed, viz., (1) samples B1–B4 exhibited higher conductivity than Z1–Z4 (2) the non-linearity conductive coefficient (αj) of ZnO-doped samples were higher than that of BST-doped samples at the same filler concentration.

As shown in Figure 3, the conductivity characteristics of both ZnO- and BST-doped samples exhibited superimposed characteristics—just like the combination of the two kinds of characteristics.

As long as the SiR composite is subjected to a DC electric field, three sources in the material, including the electrodes, fillers and impurities, will keep generating free charge carriers. Because of the electrically conductive interface region, these free charge carriers are mobile in the material and migrate to the electrode of opposite polarity instead of accumulating at the interface region. The mobility of the free charge carriers is mainly determined by the applied electric field; thus the charges are capable of migrating to the other electrode, getting neutralized with free charges of the opposite polarity or getting trapped in the trap sites. Based on the filler concentration, the transfer of free charge carriers through the bulk of the SiR composite between the electrodes is mainly determined by the conducting path created by the presence of nanoparticles in the material.

On the other hand, the non-linear conductivity properties of the material derived from the effective contact interface between the filler particles dispersed in the polymer matrix. The interfacial charges on the interface between the filler particles caused the band to bend, resulting in some sort of back-to-back Schottky barrier that rendered the non-linear conductivity behavior. 

### 2.2. Dielectric Behaviors

**Figure 4:**
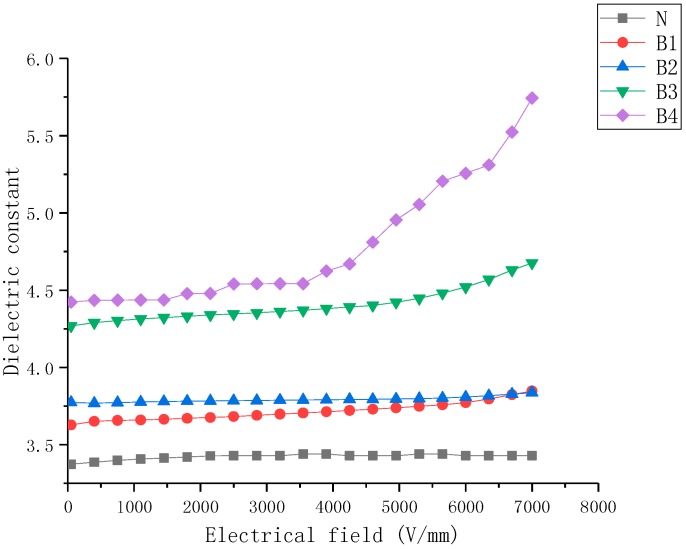
Dielectric properties of barium strontium titanate (BST)-doped silicone rubber (SiR) composites of different filler concentration at 25 °C.

**Figure 5:**
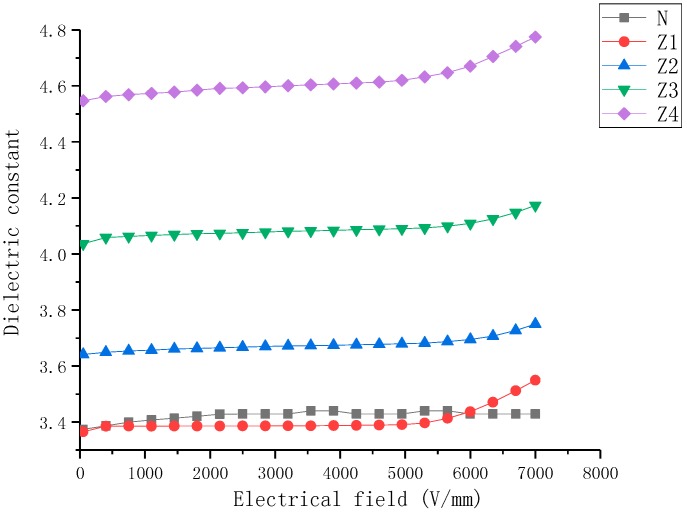
Dielectric properties of zinc oxide (ZnO)-doped silicone rubber (SiR) composites of different filler concentration at 25 °C.

**Figure 6:**
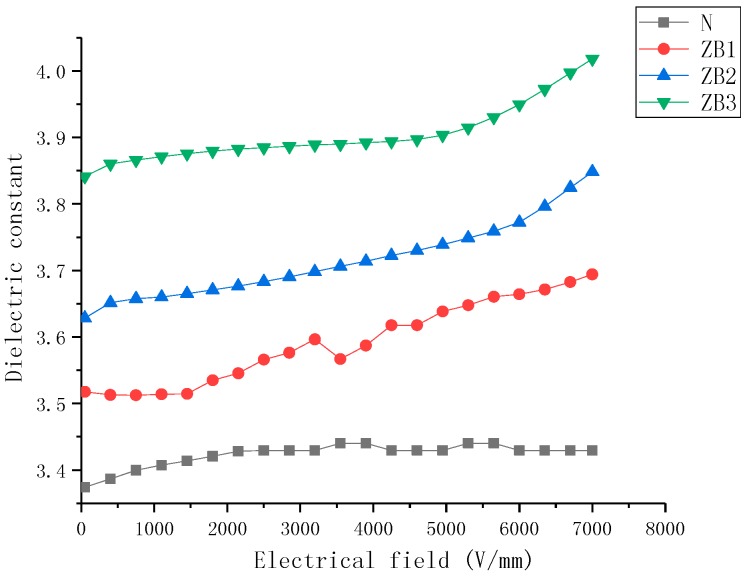
Dielectric properties of zinc oxide- (ZnO) and barium strontium titanate (BST)-doped silicone rubber (SiR) composites of different filler concentration at 25 °C.

The ε-E characteristics of nonlinear nanoparticle-doped composite samples with filler content from 5 wt% to 40 wt% are shown in Figure 4, Figure 5 and Figure 6. 

Figure 4 presents the dielectric properties of BST-doped SiR composite samples as a function of the electric field. It can be observed that the dielectric properties of BST-doped SiR samples include: (a) the permittivity of the SiR composite was enhanced due to BST fillers and the permittivity of samples increased with an increase in mass fraction of BST fillers, (b) the dielectric constant of the undoped SiR sample was relatively low and changed slightly with variation in the electric field. However, the permittivity of the BST-doped SiR samples increased with an increase in E. Samples B1–B4 exhibited nonlinear dielectric behavior, where B4 exhibited the largest nonlinearity.

Figure 5 presents the dielectric properties of ZnO-doped SiR composite samples as a function of the electric field. It can be observed that the dielectric properties of ZnO-doped SiR samples include: (a) the permittivity of the SiR composited was enhanced due to ZnO fillers (b) the permittivity of the undoped SiR sample changed slightly with variation in the electric field. Whereas the permittivity of the ZnO-doped SiR samples increased with an increase in E.

Figure 6 presents the dielectric properties of ZnO- and BST-doped SiR composite samples as a function of the electric field. It can be observed that the dielectric properties of ZB samples were similar to that of BST- and ZnO-doped samples, but ZB samples have better nonlinear properties than same mass fraction, single doped samples.

### 2.3. Loss Tangent Characteristics

**Figure 7:**
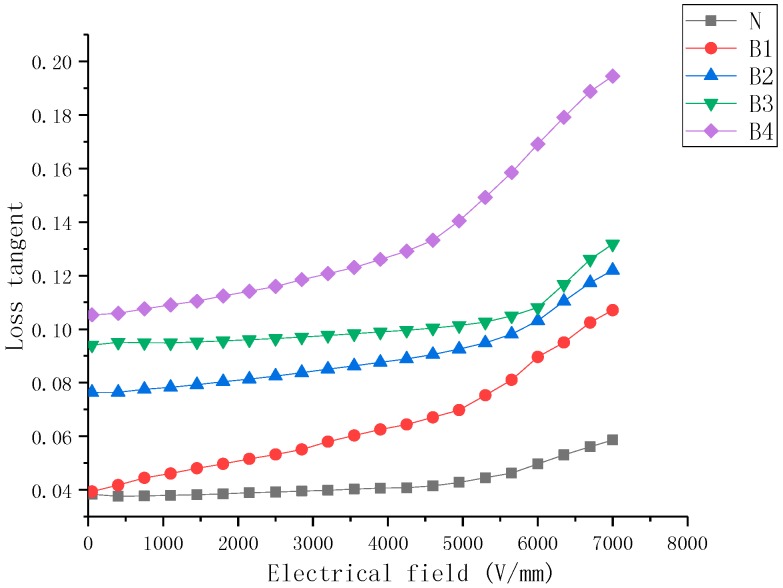
Loss tangent characteristics of barium strontium titanate (BST)-doped silicone rubber (SiR) composites of different filler concentration at 25 °C.

**Figure 8:**
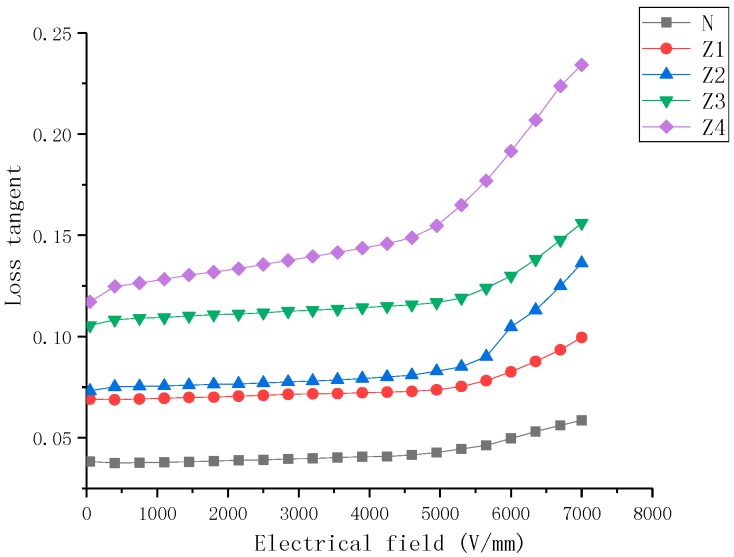
Loss tangent characteristics of zinc oxide (ZnO)-doped silicone rubber (SiR) composites of different filler concentration at 25 °C.

**Figure 9:**
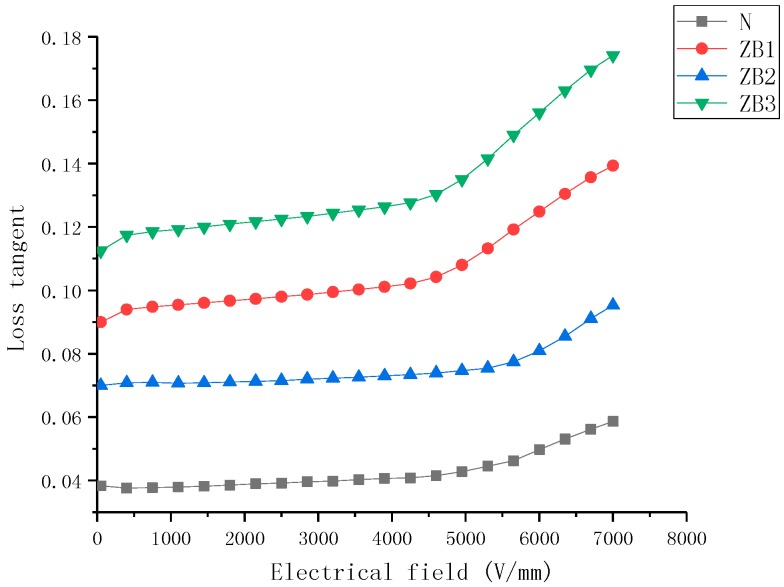
Loss tangent characteristics of zinc oxide (ZnO) and barium strontium titanate (BST)-doped silicone rubber (SiR) composites of different filler concentration at 25 °C.

Variations in the nanocomposite tanδ of samples over the electrical field strength range of 0.1–8 kV/mm are shown in Figure 7, Figure 8 and Figure 9. It can be observed that (a) for all filler loadings of BST filler, there was an increase in the tanδ values with increasing field strength, for all filler loadings of ZnO filler, there was an increase in the tanδ values with increasing field strength., (b) the tanδ of samples increased with an increase in mass fraction of BST and ZnO fillers when the concentration of fillers was 5–30% over all the studied field strength range, (c) the variation rate of the tanδ was higher at high concentration than low concentration for the same filler, (d) the tanδ values show less nonlinearity than single doped samples.

The dielectric loss tangent of the composites was mainly generated from three sources in the material including the dielectric loss of the filler and the matrix, as well as the loss caused by the interface polarization. At lower field strengths, low conductivity of ZnO and BST powder fillers resulted in low losses. With an increase in filler concentrations, the interface volume between the filler particles and the SiR matrix in the composites was also increasing, so the dielectric loss caused by interface polarization also increased. When the applied electric field exceeded the dielectric threshold field (Ec), the dielectric permittivity of ZnO filler particles increased rapidly, which enhanced the ability of the material to evacuate the space charge, resulting in a reduction of loss tangent. However, the dielectric permittivity of BST decreased as a function of the electrical field increasing, which led to an increase in the dielectric loss tangent.

### 2.4. Dielectric and Pyroelectric Response

**Figure 10:**
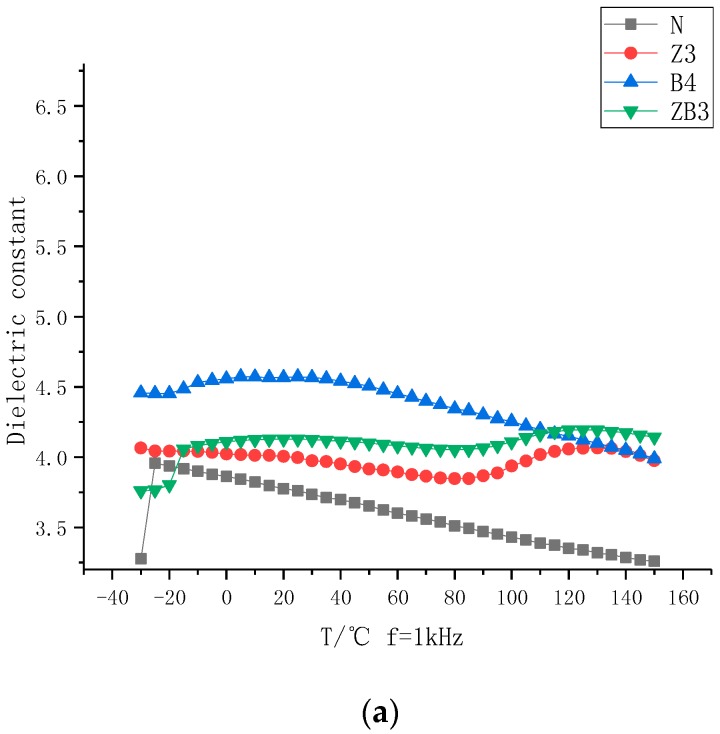

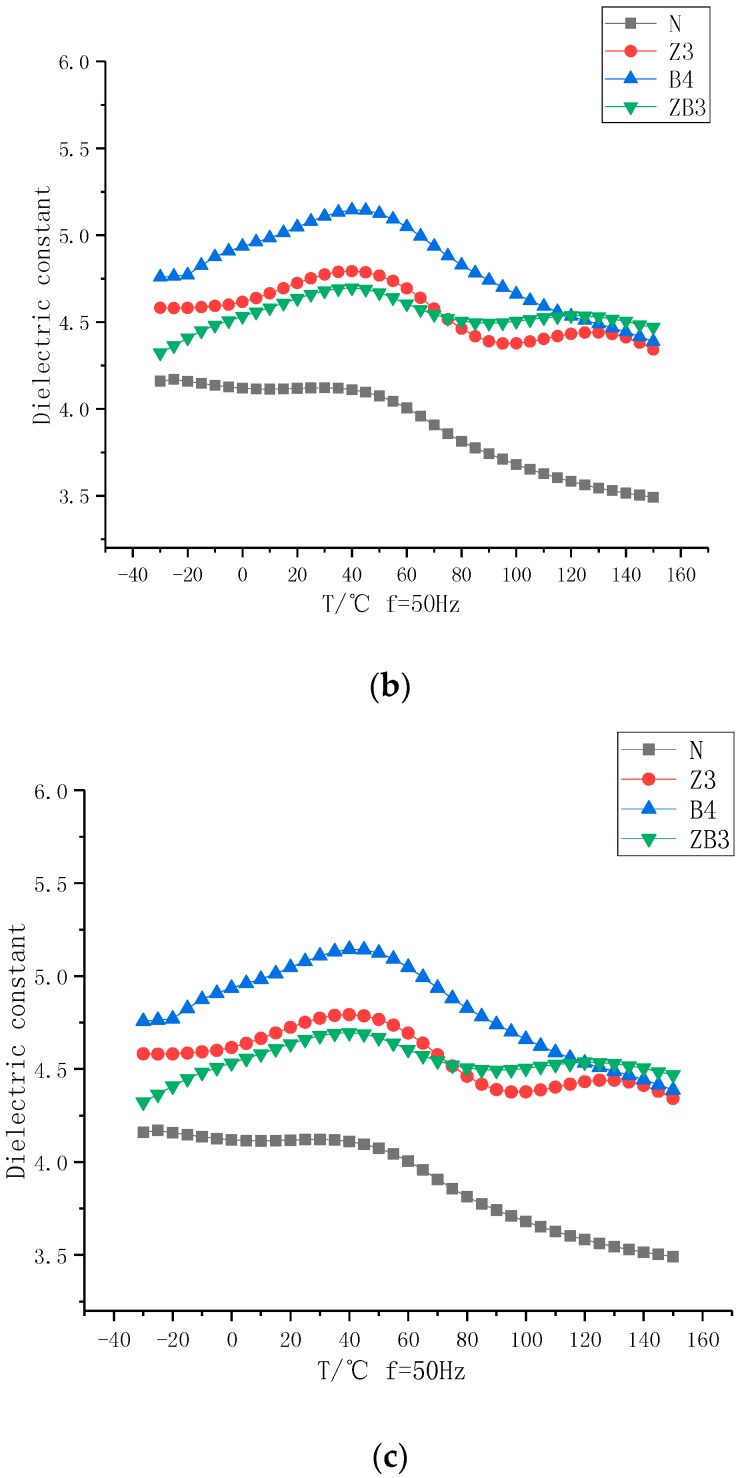
The dielectric and pyroelectric response when the test frequency is (**a**) 1 kHz (**b**) 50 Hz (**c**) 1 Hz.

Figure 10 shows that: (a) When the test frequency is 1 MHz, sample Z3 has 2 curie points around 85 °C and 130 °C, while B4 has one curie point around 20 °C and ZB3 has a combined pyroelectric response, that is, it has 3 curie points around −20 °C, 85 °C and 130 °C. (b) When the test frequency is 50 Hz, sample Z3 has 3 curie points around 40 °C, 90 °C and 130 °C, while B4 has one curie point around 40 °C and ZB3 has a combined pyroelectric response, that is, it has 3 curie points around −40 °C, 90 °C and 130 °C. (b) When the test frequency is 1 Hz, all samples have one curie point, which is 40 °C. The enormous change in ε when the frequency is close to zero Hz is a result of polarity reversal.

### 2.5. Simulation and Analysis

**Figure 11:**
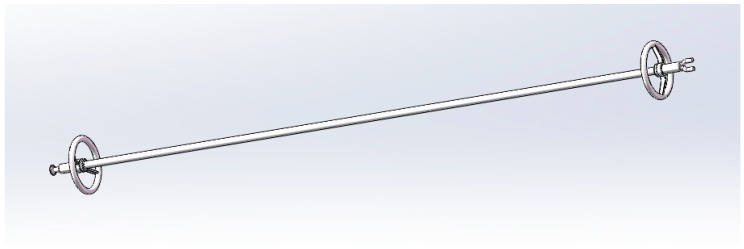
Simulation model of 220 kV AC composite insulator.

The electrical field simulation and analysis were based on a typical 330kV DC Composite insulator, the detailed parameters are shown in Figure 11:

Electrical parameters of conventional SiR materials were at a constant value. The insulator umbrella skirt and jacket were assigned a nonlinear material in this simulation model. The applied DC voltage was 220 kV. 

**Figure 12:**
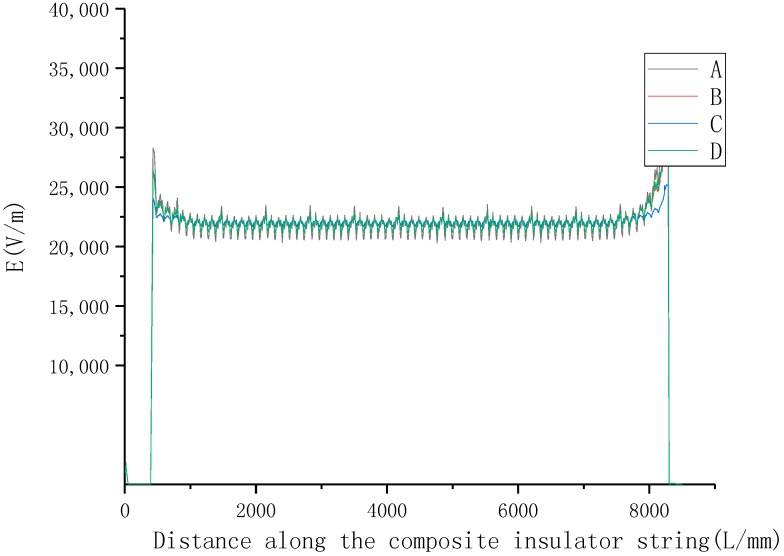
The distribution of field strength of different composite insulators along a string. (A) conventional silicone rubber (SiR) composite insulator; (B) nonlinear SiR composite B4 insulator; (C) nonlinear SiR composite Z4 insulator; (D) nonlinear SiR composite ZB3 insulator.

Curve A in Figure 12 shows the distribution of the field strength of conventional composite insulators along a string. As the curve shows, the distribution of field strength was extremely uneven due to the small longitudinal capacitance of the composite insulator. Especially, the peak electric field, which the high-voltage side of the composite insulator withstood, even exceeded 35 kV/mm.

As a comparison, the material parameters of the insulator umbrella skirt and jacket was changed with that of the SiR composites B2, Z2 and ZB3. 

For simulation’s parameter setting, we use three degree polynomial curve fitting method which uses polynomials to fitting nonlinear curves [7].

Based on the dielectric characteristic of B4 which is shown in Figure 4, the coefficients of the polynomial fitting curve used for simulation can be approximately calculated (*E*/kV/mm, 0.1 < *E* < 5 kV/mm):

(1) $$\varepsilon_{b}=t_{1}+\overset{n=3}{\underset{n=1}{\sum }}a_{n}E^{n}$$

Based on the conductivity characteristic of B4 shown in Figure 1, the coefficients of the polynomial fitting curve used for simulation can be approximately calculated (*E*/kV/mm, 0.1 < *E* < 5 kV/mm):

(2) $$\sigma_{b}=t_{2}+\overset{n=3}{\underset{n=1}{\sum }}b_{n}E^{n}$$

Based on the loss tangent characteristic of B4 shown in Figure 7, the coefficients of the polynomial fitting curve used for simulation can be calculated (*E*/kV/mm, 0.1 < *E* < 5 kV/mm):

(3) $$\tan\delta_{b}=t_{3}+\overset{n=3}{\underset{n=1}{\sum }}c_{n}E^{n}$$

In Equations (1)–(3), *t*_1_, *t*_2_, *t*_3_ are constant for the polynomial fitting curve, and *a_n_*, *b_n_*, *c_n_* are the coefficients for the electric field strength (*E*) to the nth power for the polynomial fitting curve. All of these are dimensionless constants.

The calculated *t*_1_, *t*_2_, *t*_3_ and *a_n_*, *b_n_*, *c_n_* (*n* = 1, 2, 3) for B4 are shown in Table 1.

Same method is used in simulation for Z4 and ZB3. A finite element simulation was used to obtain a convergence solution. The electric field distribution energy error was less than 1% after eleven steps iterative solving with electrostatic field solver. The electrical field distributions of B4, Z4 and ZB3 are shown by Figure 12B–D. It was shown that nonlinear material can cause the electrical field to be even, and the combination of ZNO and BST is better than any one of them. The percentage of withstand voltage is shown in Table 2.

Results demonstrated that nonlinear SiR materials with adaptive electric field properties were capable of homogenizing an uneven electrical field distribution. The improvement in electric field distribution mainly came from two aspects: (a) nonlinear SiR material had higher permittivity and conductivity than unfilled SiR under the field strength of the actual working condition. Previous investigation had verified that high permittivity conduced a reduction in high capacitive partial pressure, as well as a reduction in resistive partial pressure and the dissipation of accumulated charge. (b) The conductivity of the material had the characteristic of self-adapting with the electric field. When the electric field became higher, the rise in conductivity also enhanced the partial pressure capability of the material.

The result obtained by the simulation demonstrated that nonlinear SiR material could significantly mitigate the potential gradient at the high-voltage side of the composite insulator, while it had less of an effect on the central field strength. Therefore, reducing the length of the nonlinear material of the insulator, and using it only at the high-voltage side instead of throughout the string, can be considered. As the ratio of the permittivity of the two contact materials significantly affects the field strength at the interface, it can be considered that the entire composite insulator was divided into segments and the BST doping amount of each segment gradually decreased, so that the permittivity was gradually changed to the ordinary value to avoid the field distortion.

## 3. Discussion

Previous studies have shown that the nonlinear dielectric properties of ZnO-doped SiR composites are mainly due to the existence of three relaxation processes in the composites: α relaxation caused by long SiR segments, β-relaxation caused by the polar molecule and IDE relaxation caused by the filler particles [8,9]. These three relaxation processes together determine the dielectric properties of the composite. When the filler loading was less than 10%, the relaxation activation energy could be determined by the combination of ZnO particles and the SiR matrix, which indicated that the dielectric properties of the composite were also determined by the dielectric properties of both filler and matrix [10]. When the filler loading was more than 20%, the activation energy was close to the grain boundary activation energy of ZnO varistor ceramics. In such a condition, the dielectric properties of the composites were mainly controlled by ZnO filler particles, which would exhibit obvious nonlinearity of dielectric permittivity as a function of the electrical field [11].

BaTiO_3_ is usually in the form of ferroelectric ceramic particles, and Ba_0.6_Sr_0.4_TiO_3_ was shown to be in the paraelectric phase at room temperature. In a previous study, the ‘distorted nano-region’ model was used to study the ferroelectric properties of Ba_0.6_Sr_0.4_TiO_3_ ceramic [12]. ‘Polar nano-region’ is related to dielectric nonlinearity of BST in the paraelectric state, which is defined as a nanometer scale region with parallel oriented spontaneous polarization, which consists of each other acting as a giant dipole with slow relaxation frequency. The ‘distorted nanoregion’ potentially makes it difficult to form a ‘polar nano-region’ or baffle its response to an external field vector [10]. Therefore, the doped composite of samples B1 to B4 showed a similar performance compared to BST ceramics.

In the case of a SiR composite system, it has been reported that an increase in dielectric permittivity presumably resulted from an intervention in the mobility of SiR chains in the bulk of the SiR composite [13,14]. Previous documents reported that a constraint in the mobilities of polymer chains in a SiR composite was probably due to the interaction process between filler particle surfaces and polymer chains [15,16].

Based on the interface model, two nano-layers around the particle formed as a result of the polymer-nanoparticle interactions. The first nano-layer which was closest to the nanoparticle surface was presumably firmly tied to the surface which caused the polymer chains close to the first nano-layer to become highly immobile. The second polymer was a lot thicker than that of the first layer and the polymer chains were loosely bound to the second layer. It was the dynamics of these interface polymer regions, concerning the filler particle concentrations, which resulted in a variation in the dielectric and conductive properties [17,18]. It is essential to mention that the filler dispersion in the SiR matrix was a precondition of the validity of the interface model used here. The formation of the two interface layers and the unique properties of each of the layers are valid only if the filler particles were dispersed evenly in the SiR matrix during the preparation process. According to the permittivity characteristics and characterization of samples obtained, it can be presumed that the nanoparticles have been well dispersed in the SiR matrix.

At the low filler loadings of 0–5 wt%, the influence of chain immobility (caused by the first layer of interface) and chain entanglements [19] would restrict the mobility of dipolar groups in the SiR system leading to its conductivity and loss tangent to be close to that of the unfilled SiR composites. With increasing filler loadings and electrical field, the permittivity of the filler started to have more and more influence on that of the nanocomposites [17,18]. At the high filler loadings, the permittivity of the SiR system is mainly determined by the combined interaction of chain immobilization and the rate of permittivity enhancement of the material concerning the filler concentration. In a previous publication, Tanaka et al. [20] put forward a multi-core model to explain the dielectric characteristics investigated for nanocomposite systems. Compared to four layers of the interface mentioned in the multi-core model, the model used in this study only mentioned two layers. Some of the interaction mechanisms responsible for the nanocomposite dielectric characteristics as per the 2-layer model in this study can be connected to the multi-core model. The formation of the tightly bound first layer and the loose second layer in the present model can be seen as the second and third layers of the multi-core model.

## 4. Materials and Methods

### 4.1. Material Processing and Sample Preparation

The silicone rubber with different fillers was prepared as follow:

1940 g of methyl vinyl silicone rubber (molecular weight 600,000, vinyl mass fraction 0.03%) and 60 g of vinyl silicone oil (12%, viscosity 4000 mPa·s) were used to obtain 2 kg of rubber. Four grams of Zinc stearate and 100 g of silazane were dissolved in 40 g water. Then, 700 g of white carbon black and 2 kg of the prepared rubber were added separately. The mixture was stirred in a kneading machine for 1 h at room temperature. Afterwards, the kneading machine was set to 180 °C and start to vacuum at 90 °C. The mixture was stirred at 180 °C for another 1 h and cooled down to obtain solid mixture A. According to the weight of mixture A, 5.2 g of vinyltriethoxysilane was add to 2080 g of aluminium hydroxide to mix with 260 g of talcum powder to obtain powder mixture B, which was used to improve the dispersion of nonlinear fillers in the rubber compound and the anti-tracking properties of material. Mixture A and B were mixed and stirred in kneading machine for 1 h at room temperature to obtain the stable and uniform rubber compound.

Two kinds of nonlinear power were selected as the fillers of silicone rubber composites which were BST (Ba_x_Sr_1−x_TiO_3_ x = 0.6, 200 nm, sintered at 1300 °C, shanghai dianyang Advanced Technology Co., China) and ZnO (40 nm, Nanjing emperor nano material Co., Nanjing, China). The prepared power was evenly added to the silicone rubber with open mill, and the mass fractions of fillers ranged from 5% to 40%. The rubber was compressed into tablets to get silicone rubber samples. In this way, fourteen test groups were set. Each test group contained 5 pieces of silicone rubber samples with the same composition. The samples designation and contents are shown in Table 3.

### 4.2. Characterization

The crystal structure of fillers and samples were analyzed using X-ray diffraction (XRD, D/max-2500/PC, Rigaku Co., Tokyo, Japan), at a scanning speed of 10° (angle degree)/min in steps of 0.02°. The microstructure of the samples was measured with a scanning electron microscope (SEM) (Nova Nano SEM450, FEI, FEI Co., Hillsboro, OR, America). 

**Figure 13:**
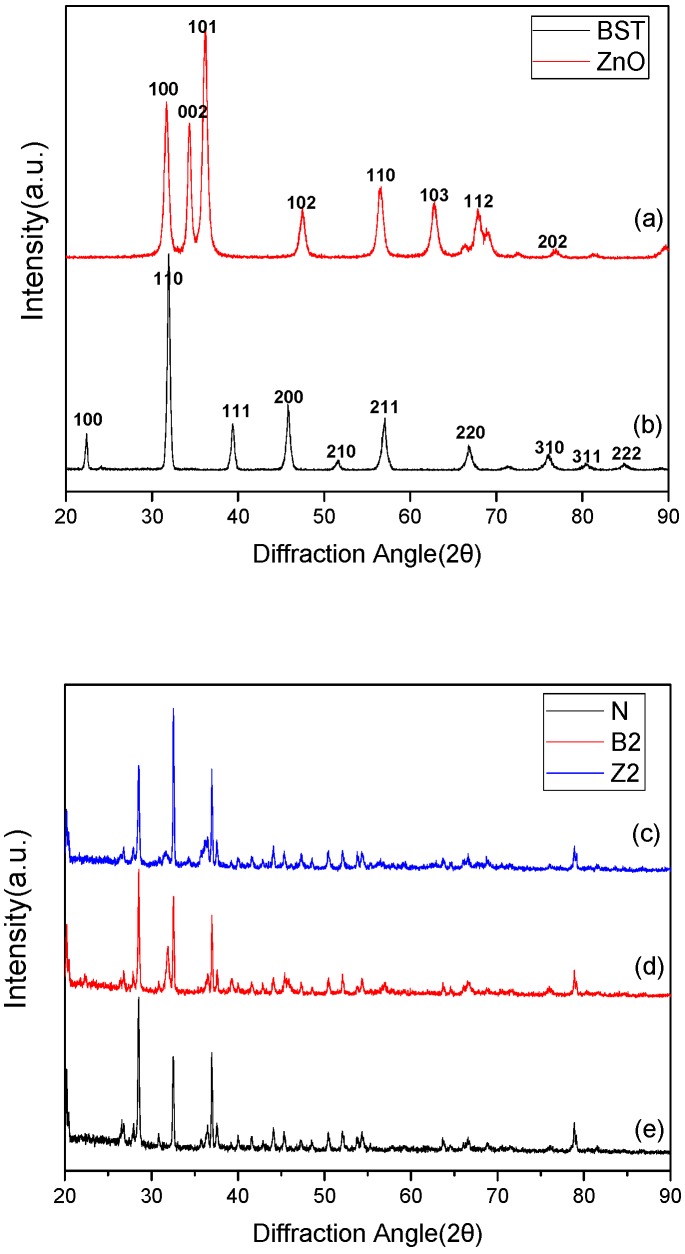
X-ray diffraction (XRD) patterns of filler particles and silicone rubber (SiR) samples. (**a**) zinc oxide (ZnO) powders; (**b**) barium strontium titanate (BST) powders; (**c**) sample N; (**d**) sample B2; (**e**) sample Z2.

Figure 13 shows the XRD patterns of filler particles and SiR samples. Compared with the PDF card, a figure of the powder was: BST, perovskite structure; ZnO, hexagonal wurtzite structure. The peaks of the two materials were sharp which indicated the desirable crystallinity of BST and ZnO particles. It can be seen from Figure 13 that sample B2 contained BST particles and Z2 contained ZnO particles, and in the doping process, no crystal change of inorganic filler took place.

**Figure 14:**
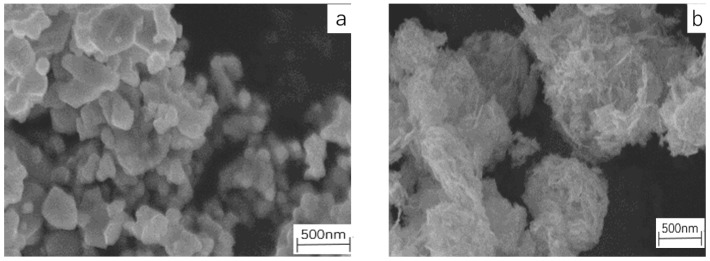

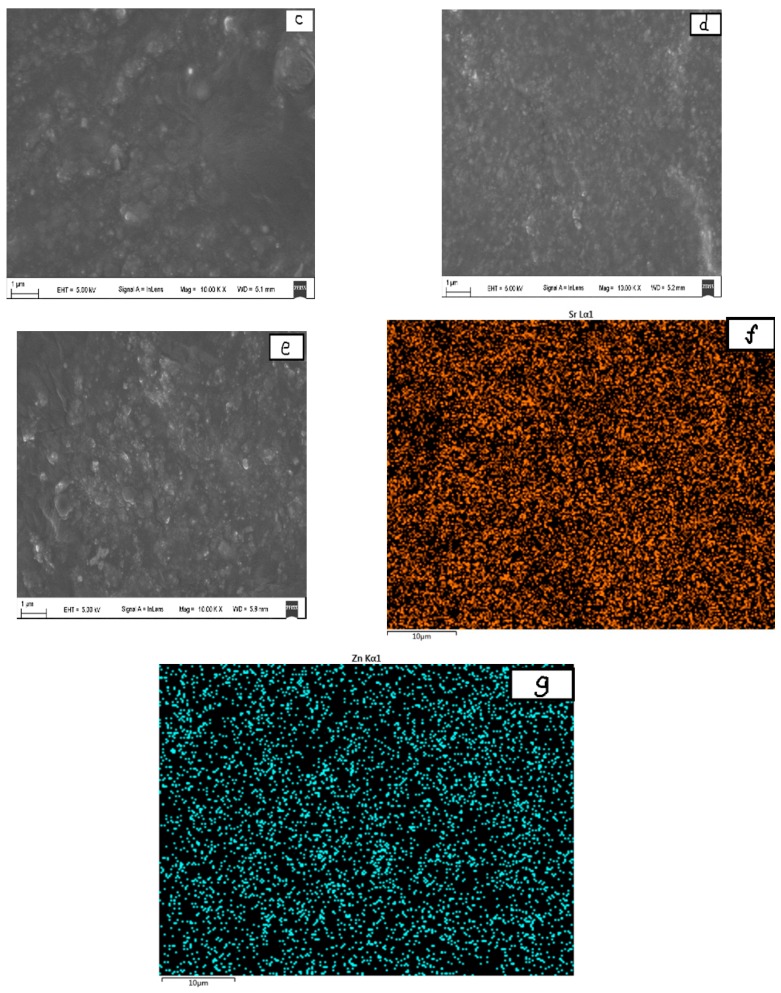
Scanning electron microscope (SEM) images showing the dispersion of silicone rubber (SiR) samples and the microstructure of the SiR composites and filler particles. (**a**) barium strontium titanate (BST) powders; (**b**) zinc oxide (ZnO) powders; (**c**) sample B4; (**d**) sample Z4; (**e**)sample ZB2 (**f**) energy dispersive spectrometer (EDS) map scanning images of Sr of B2; (**g**) EDS map scanning images Zn of Z2.

Figure 14a–f shows the SEM morphology of the BST and ZnO ceramic powers. As shown in (a), the average diameter of the BST particles prepared in this study was about two hundred nanometers. A 2–3 um powder particle comprised dozens of crystal grains, which required a dispersing process to control the size of fillers during the preparation of samples. Figure 14b is the SEM images of ZnO powders which could been seen as a hexagonal wurtzite structure. Powder particles were easy to agglomerate which indicated that a dispersing process was needed during the preparation. Figure 14c–e shows the SEM morphology of samples B2, Z2 and ZB2. In these pictures, some tiny grain particles were found in the surface of samples. The dispersion of these grain particles represented the dispersion of fillers in the SiR matrix. Figure 14f,g are the energy dispersive spectrometer (EDS) map scanning images of Ti of B2 and Zn of Z2, which shows that the Zn element distribution in sample Z2 and the Ti distribution in sample B2 were uniform. The uniform dispersion of Ti and Zn indicated that the inorganic filler BST and ZnO were evenly doped in the mixed silicone rubber and the composite material was homogenous. The contact interface between SiR matrix and filler particles was favorable.

### 4.3. Test System and Experimental Details

The nonlinear electrical characteristics and dielectric and pyroelectric response of composites were analyzed by a broadband dielectric analyzer (Concept 80 Novocontrol GmbH, Montabaur, Germany). The sample was sandwiched between two 20 mm gold plated electrodes tested from AC 0.02 kV to 1.4 kV and −30° to 150°, and then the broadband dielectric analyzer imposed a relatively weak signal at a certain frequency on the sample. Combined with its geometric parameters, the electrical parameter of the sample could be calculated. All the factors including test temperature, frequency and DC bias voltage could be flexibly adjusted. The DC bias voltage range applied to the sample was 0–2000 V, AC small signal frequency for the frequency (50 Hz), the effective value of 1 V. All the measurements were made at room temperature.

In this study, characteristics of SiR composites at different filler concentrations by weight were evaluated. The characterization of phase and microstructure of SiR composites was made by an energy dispersive spectrometer (EDS), X-ray diffraction (XRD) and scanning electron microscope (SEM). Furthermore, the evaluations of electrical properties were made by performing conductivity (ρ), dielectric constant (εr) and loss tangent (tanδ) measurements.

## 5. Conclusions

SiR-nonlinear filler nanocomposite systems were examined for their dielectric behaviors in this study primarily for two reasons, (a) to validate the prospect of tailoring the electrical properties of SiR nanocomposite systems with BST and ZnO fillers, and (b) to characterize and understand the dielectric properties of this system. Results of this study corroborated some of the interesting dielectric characteristics based on the different electrical behaviors of BST and ZnO samples, the electrical behaviors of SiR composites can be tailored to obtain insulating material with suitable properties for a specific situation. Still, further experiments were required to verify the validity of the multi-core model and the interface model, used here to explain the dielectric properties of the materials.

## Figures and Tables

**Table 1:** Parameters of the fitting function of B4.

**Parameter**	***t*_1_**	***a*_1_**	***a*_2_**	***a*_3_**
value	4.4465	0.0081	8 × 10^−4^	1 × 10^−4^
**Parameter**	***t*_2_**	***b*_1_**	***b*_2_**	***b*_3_**
value	1 × 10^−8^	3 × 10^−10^	3 × 10^−11^	2 × 10^−12^
**Parameter**	***t*_3_**	***c*_1_**	***c*_2_**	***c*_3_**
value	0.1039	0.0016	1 × 10^−4^	1 × 10^−5^

**Table 2:** The vertical insulation distances of composite insulators of different materials bearing different voltages.

	Percentage of Withstand Voltage (%)	20%	40%	60%	80%
Conventional SiR	Percentage of length of string (%)	23.4%	42.1%	60.8%	79.6%
Nonlinear SiR	Percentage of length of string (%)	20.1%	39.6%	59.6%	82.4%

**Table 3:** Samples prepared in this study.

Designation	ZnO Content (wt.%)	BST Content (wt.%)
Z1	5	-
Z2	10	-
Z3	20	-
Z4	30	-
B1	-	5
B2	-	10
B3	-	15
B4	-	20
ZB1	2.5	2.5
ZB2	5	5
ZB3	10	10
N	-	-
